# Risk Factors for Postoperative Complications in Different Fusion Surgical Approaches for Lumbar Degenerative Diseases

**DOI:** 10.3390/jcm15114195

**Published:** 2026-05-29

**Authors:** Zhenbiao Zhu, Anwu Xuan, Cheng Xu, Chaofeng Wang, Qing He, Liang Tang, Dike Ruan

**Affiliations:** 1 The Second School of Clinical Medicine, Southern Medical University, No. 1023, South Shatai Road, Baiyun District, Guangzhou 510515, China; bong1976@163.com (Z.Z.); m13575139792@163.com (L.T.); 2 Department of Orthopedics, The Sixth Medical Center of PLA General Hospital, Beijing 100048, China; xuanawcyxuan@163.com (A.X.); xuchengngh@163.com (C.X.); heqingngh@163.com (Q.H.); 3 Department of Orthopaedics, Haikou Research Institute of Xiangya School of Medicine, Central South University, Haikou 570208, China; 4 Department of Orthopedics, General Hospital of Northern Theater Command, Shenyang 110015, China; 5 Department of Orthopedics, Xi’an Honghui Hospital, Xi’an 710054, China; fengwcf2007@163.com

**Keywords:** lumbar degenerative diseases, lumbar fusion, postoperative complications, risk factors, cage migration, adjacent segment degeneration

## Abstract

**Objective:** Posterior lumbar interbody fusion (PLIF), posterolateral fusion (PLF), and Hybrid fusion are widely used fusion procedures for lumbar degenerative diseases (LDDs). Postoperative complications dominated by cage migration (CM) and adjacent segment degeneration (ASD) remain major challenges. This study aimed to identify and compare the independent risk factors for CM and ASD in PLIF, PLF, and Hybrid fusion, so as to provide evidence-based references for preoperative evaluation, surgical selection, and complication prevention in clinical practice. **Methods:** A retrospective cohort study was conducted in patients who underwent PLIF, PLF, or Hybrid fusion for LDDs at our institution. Demographic data (age, gender, and body mass index [BMI]), lifestyle factors (smoking and insobriety), comorbidities (hypertension, diabetes, hyperuricemia, osteoporosis, and hypoalbuminemia), surgical parameters (operative time, intraoperative blood loss, fusion segments, and lumbar lordosis angle), radiological indices (Pfirrmann grading of intervertebral disc degeneration and relative disc height), and biological markers (C-reactive protein/lymphocyte ratio [CLR], procalcitonin [PCT], and serum amyloid A [SAA]) were collected. Patients were stratified into complication and non-complication groups based on the occurrence of CM or ASD. Univariate and binary logistic regression analyses were performed to determine independent risk factors for postoperative complications. **Results:** A total of 203 patients were enrolled, including 80 cases with complications in the PLIF group, 64 in the Hybrid group, and 59 in the PLF group. No significant differences were noted in the distribution of complication types among the three groups (*p* = 0.179). Univariate analysis revealed that BMI, osteoporosis, the Pfirrmann grading of superior adjacent disc degeneration, lumbar lordosis angle, operative time, and intraoperative blood loss were significantly associated with postoperative complications across all three surgical groups (*p* < 0.05). Binary logistic regression analysis confirmed that elevated BMI (PLIF: OR = 1.18, 95%CI: 1.05–4.38; PLF: OR = 1.19, 95%CI: 0.76–2.18; Hybrid: OR = 1.14, 95%CI: 1.07–2.54), osteoporosis (PLIF: OR = 6.86; PLF: OR = 7.62; Hybrid: OR = 5.62), advanced superior adjacent disc degeneration (PLIF: OR = 8.04; PLF: OR = 4.49; Hybrid: OR = 2.87), prolonged operative time, and increased intraoperative blood loss were independent risk factors for postoperative complications. In contrast, age, gender, smoking, insobriety, hypertension, diabetes, CLR, PCT, and SAA were not identified as risk factors (p* > 0.05). **Conclusions:** Elevated BMI, osteoporosis, pre-existing superior adjacent disc degeneration, prolonged operative time, and increased intraoperative blood loss are shared independent risk factors for CM and ASD following PLIF, PLF, and Hybrid fusion for LDDs. Targeted interventions addressing these factors may reduce postoperative complication rates and improve patient outcomes.

## 1. Introduction

Lumbar degenerative diseases (LDDs), including lumbar disc herniation, lumbar spinal stenosis, and degenerative spondylolisthesis, represent a leading cause of chronic low back pain and neurological deficits in the aging population [1,2]. With the global trend of population aging, the incidence of LDDs has risen dramatically, imposing a substantial burden on healthcare systems worldwide. Posterior lumbar interbody fusion (PLIF) and posterolateral fusion (PLF) are well-established surgical interventions for refractory LDDs, as they effectively decompress neural structures, restore spinal alignment, and enhance segmental stability [3,4,5,6]. PLIF, in particular, offers the advantages of reconstructing disc height, maintaining sagittal balance, and achieving robust interbody fusion [5,6]. The Hybrid fusion technique, which combines distal segment PLIF and proximal segment PLF, has also gained popularity for its tailored approach to multi-segment LDDs [7,8].

Despite high success rates, postoperative complications remain a major concern. Cage migration (CM) and adjacent segment degeneration (ASD) are two distinct but clinically important complications after lumbar fusion. CM is an acute mechanical complication related to implant stability, while ASD is a chronic degenerative process related to biomechanical stress redistribution. Both may cause recurrent symptoms and revision surgery. Numerous studies have investigated the risk factors for postoperative complications following lumbar fusion [9,10]. Proposed risk factors include demographic characteristics (e.g., age, gender, and BMI), comorbidities (e.g., osteoporosis and diabetes), surgical parameters (e.g., fusion segments and operative time), radiological features (e.g., preoperative disc degeneration and lumbar lordosis angle), and biological markers (e.g., C-reactive protein [CRP] and serum albumin) [2,11]. However, the existing literature predominantly focuses on single surgical modalities (e.g., PLIF or TLIF [transforaminal lumbar interbody fusion]) [7,8], with limited comparative analyses of PLIF, PLF, and Hybrid fusion. Furthermore, the findings regarding risk factors such as age and gender remain controversial, highlighting the need for a comprehensive, comparative study across different fusion techniques.

Nutritional status and systemic inflammatory responses also play pivotal roles in postoperative recovery. Hypoalbuminemia (serum albumin < 3.5 g/dL), a marker of malnutrition, has been linked to increased wound complications, prolonged hospital stays, and higher readmission rates following spinal surgery [1,12,13,14]. Inflammatory biomarkers, including the CRP/lymphocyte ratio (CLR), have emerged as promising predictors of surgical site infection, with superior diagnostic accuracy compared with individual markers. However, the association between these biomarkers and complications such as CM and ASD remains poorly understood.

Against this backdrop, the present study aimed to systematically compare the risk factors for CM and ASD following PLIF, PLF, and Hybrid fusion for LDDs. By integrating demographic, clinical, radiological, and biological data, we sought to identify shared and modality-specific risk factors, thereby providing a theoretical basis for optimizing preoperative assessment, surgical planning, and postoperative management.

## 2. Materials and Methods

### 2.1. Study Population

A retrospective cohort study was performed in patients who underwent PLIF, PLF, or Hybrid fusion for LDDs between January 2018 and December 2023. The inclusion criteria were: (1) confirmed diagnosis of LDD (lumbar disc herniation, lumbar spinal stenosis, or degenerative spondylolisthesis) based on clinical and radiological findings; (2) primary fusion surgery using PLIF, PLF, or Hybrid technique; (3) complete preoperative, intraoperative, and postoperative data; and (4) minimum 2-year consistent follow-up with full imaging and clinical records. Exclusion criteria included: (1) prior spinal surgery; (2) traumatic, tumorous, or infectious spinal diseases; (3) incomplete follow-up or missing key data; and (4) severe systemic diseases affecting outcomes (e.g., end-stage renal disease, liver cirrhosis, or metastatic cancer).

### 2.2. Data Collection

Demographic and clinical data collected included age, gender, smoking status (≥10 cigarettes/day), excessive alcohol drinking (≥50 mL/day of liquor), body mass index (BMI), bone mineral density (BMD), and comorbidities (hypertension, diabetes mellitus, hyperuricemia, osteoporosis, and hypoalbuminemia). Surgical parameters included operative time, intraoperative blood loss, average postoperative drainage volume, number of fused segments, relative intervertebral disc height, Pfirrmann classification of disc degeneration, lumbar lordosis (LL) angle, primary symptom (low back pain or leg pain), and duration of symptoms. Radiological assessments included BMD measurement via dual-energy X-ray absorptiometry (DXA) and evaluation of intervertebral disc degeneration using the Pfirrmann classification system (grades I–V) [15]. Clinical outcome measures included the Visual Analog Scale (VAS) for pain [16], the Japanese Orthopaedic Association (JOA) score [17], the Short-Form 36 (SF-36) score [18], and the Oswestry Disability Index (ODI) [19]. Biological markers analyzed included preoperative C-response protein-to-lymphocyte count ratio (CLR), procalcitonin (PCT), and serum amyloid A (SAA). Related risk factors include hypertension, diabetes, coronary heart disease, hypoproteinemia, smoking, drinking, hyperuricemia, overweight, osteoporosis, etc.

### 2.3. Surgical Technique

**Figure 1:**
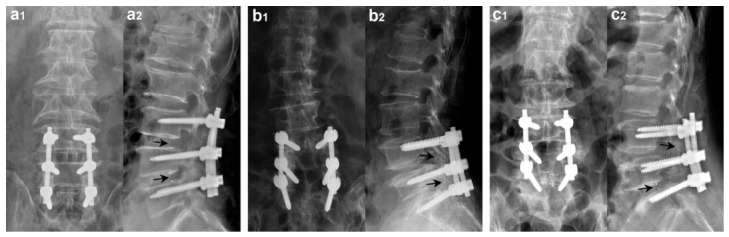
Radiographic images of 2-level lumbar fusion. (**a1**,**a2**) Postoperative anteroposterior and lateral X-ray images of posterior lumbar interbody fusion (PLIF). (**b1**,**b2**) Postoperative anteroposterior and lateral X-ray images of posterior lumbar fusion (PLF). (**c1**,**c2**) Postoperative anteroposterior and lateral X-ray images of hybrid surgery combining distal PLIF with proximal PLF.

All surgical procedures were performed by experienced spine surgeons in accordance with standardized protocols [20]. PLIF involved posterior midline incision, laminectomy, decompression of neural structures, discectomy, endplate preparation, interbody cage insertion, and pedicle screw fixation. PLF included posterior midline incision, exposure of the transverse processes and facet joints, decortication, bone grafting, and pedicle screw fixation. Hybrid fusion combined PLIF at the distal segments and PLF at the proximal segments to balance stability and tissue preservation (Figure 1).

### 2.4. Statistical Analysis

All statistical analyses were performed using IBM SPSS Statistics 21.0 (IBM Corp., Armonk, NY, USA) (Supplemental Table S1). Continuous variables were compared by the *t*-test or Mann–Whitney U test and categorical variables by the χ^2^ test. Multicollinearity was tested using the variance inflation factor (VIF); a VIF < 2 was defined as no significant collinearity. Binary logistic regression was performed. Model performance was evaluated using AUC, sensitivity, and specificity. *p* < 0.05 was considered significant.

## 3. Results

A total of 436 patients were enrolled in this study, including 160 in the PLIF group (80 with complications and 80 without complications), 152 in the PLF group (59 with complications and 93 without complications), and 144 in the Hybrid group (64 with complications and 80 without complications). The overall cohort had a mean age of 62.5 ± 7.2 years, with 189 males (43.3%) and 247 females (56.7%). The median follow-up duration was 28.6 ± 6.3 months.

### 3.1. Distribution of Postoperative Complications

CM and ASD were the main postoperative complications (Table 1). In the PLIF group, 15 patients (18.8%) developed CM, and 65 patients (81.2%) developed ASD. Since no interbody cage was implanted in the PLF group, only ASD occurred in 59 cases (100%). In the Hybrid group, 6 cases (9.4%) presented with CM, and 58 cases (90.6%) presented with ASD. There were no significant differences in the distribution of complication types among the three groups (*p* = 0.179).

### 3.2. Comparison of Baseline Characteristics

In all three surgical groups, the complication group had significantly higher BMI, higher osteoporosis prevalence, and lower bone mineral density (BMD) than the non-complication group (all *p* < 0.01). The mean BMI values in the complication vs. non-complication groups were 24.97 ± 3.12 vs. 22.35 ± 2.89 kg/m^2^ (PLIF), 24.89 ± 3.45 vs. 22.23 ± 3.19 kg/m^2^ (PLF), and 24.88 ± 2.49 vs. 21.95 ± 3.01 kg/m^2^ (Hybrid). The prevalence rates of osteoporosis were 30.0% vs. 10.0% (PLIF), 28.8% vs. 11.8% (PLF), and 25.0% vs. 7.5% (Hybrid). The mean BMD values were −2.34 ± 0.93 vs. −0.56 ± 0.73 (PLIF), −2.32 ± 0.65 vs. −0.66 ± 0.88 (PLF), and −2.30 ± 0.86 vs. −0.77 ± 0.38 (Hybrid).

Hypoalbuminemia was significantly more frequent in the PLIF complication group (18.8% vs. 10.0%; *p* = 0.02), while no significant differences were found in the PLF (23.7% vs. 19.4%; *p* = 0.52) and Hybrid (23.4% vs. 18.7%; *p* = 0.491) groups. No significant intergroup differences were observed in age, gender, smoking, alcohol abuse, hypertension, diabetes, and hyperuricemia (all *p* > 0.05) (Supplemental Table S2).

### 3.3. Comparison of Clinical and Radiological Indicators

For upper adjacent disc degeneration assessed by Pfirrmann grade, the grade V proportion was significantly higher in the PLIF complication group (18.8% vs. 3.8%; *p* < 0.05), whereas no significant differences were observed in the PLF (*p* = 0.08) and Hybrid (*p* = 0.307) groups. The Pfirrmann grade of the lower adjacent segment was significantly higher in the complication group across all three surgical approaches (all *p* < 0.05). The rates of grade V degeneration were 25.0% vs. 12.5% (PLIF), 18.6% vs. 8.2% (PLF), and 18.8% vs. 7.5% (Hybrid). Lumbar lordosis (LL) angle was significantly smaller in the complication group: PLIF (39.6 ± 10.9° vs. 44.5 ± 9.2°), PLF (37.6 ± 6.7° vs. 42.6 ± 8.9°), and Hybrid (39.5 ± 7.9° vs. 47.4 ± 8.9°) (all *p* < 0.01) (Supplemental Table S3).

### 3.4. Comparison of Intraoperative Indicators

Operative time was significantly prolonged in the complication group in all three groups (all *p* < 0.01): PLIF (230.0 ± 45.2 vs. 219.0 ± 49.4 min), PLF (176.1 ± 36.47 vs. 158.1 ± 34.68 min), and Hybrid (205.6 ± 40.5 vs. 180.3 ± 33.6 min). Intraoperative blood loss was also significantly increased in the complication group (all *p* < 0.05): PLIF (399.17 ± 94.7 vs. 350.17 ± 85.7 mL), PLF (351.1 ± 90.5 vs. 316.8 ± 86.8 mL), and Hybrid (408.6 ± 89.2 vs. 370.75 ± 95.24 mL). Detailed intraoperative parameters are shown in Table 2.

### 3.5. Comparison of Biological Markers and Clinical Outcome Scores

The CLR was significantly elevated in the complication group across all three surgical cohorts (all *p* < 0.01). PCT and SAA showed no significant differences between complication and non-complication groups (all *p* > 0.05) (Table 3). Preoperative ODI, VAS, and JOA scores were significantly different between complication and non-complication groups in all three approaches (all *p* < 0.01). The SF-36 score only showed a significant difference in the PLIF group (21.04 ± 5.17 vs. 23.03 ± 5.53; *p* < 0.01) (Table 4).

### 3.6. Multivariate Logistic Regression Analysis

Elevated BMI, osteoporosis, advanced adjacent segment disc degeneration, prolonged operative time, and increased intraoperative blood loss were common independent risk factors for postoperative complications across three fusion approaches (Supplemental Tables S4–S6).

Each 1-unit increase in BMI elevated complication risk by 18% in PLIF (OR = 1.18, 95%CI: 1.05–4.38, *p* < 0.05), by 19% in PLF (OR = 1.19, 95%CI: 1.06–2.18, *p* < 0.05), and by 14% in Hybrid fusion (OR = 1.14, 95%CI: 1.07–2.54, *p* < 0.05). Osteoporosis increased the complication risk significantly: PLIF (OR = 6.86, 95%CI: 3.25–14.48, *p* < 0.001), PLF (OR = 7.62, 95%CI: 3.81–15.24, *p* < 0.001), and Hybrid (OR = 5.62, 95%CI: 2.78–11.35, *p* < 0.001). Advanced upper adjacent disc degeneration was also an independent risk factor: PLIF (OR = 8.04, 95%CI: 4.12–15.68, *p* < 0.001), PLF (OR = 4.49, 95%CI: 2.25–8.96, *p* < 0.001), and Hybrid (OR = 2.87, 95%CI: 1.43–5.76, *p* < 0.05).

Lumbar lordosis angle was an independent risk factor for PLIF (OR = 1.85, 95%CI: 1.16–3.42, *p* = 0.01) and Hybrid fusion (OR = 1.58, 95%CI: 1.08–2.78, *p* = 0.02). Age, gender, smoking, alcohol abuse, hypertension, diabetes, CLR, PCT, SAA, ODI, JOA, VAS and SF-36 were not independent risk factors (all *p* > 0.05).

## 4. Discussion

Lumbar fusion surgery is the cornerstone of treatment for refractory LDDs, yet postoperative complications such as CM and ASD continue to limit its clinical efficacy [21,22]. The present study represents one of the few comparative analyses of risk factors for complications across PLIF, PLF, and Hybrid fusion techniques, providing novel insights into the shared and modality-independent risk factors for CM and ASD. Our key findings indicate that elevated BMI, osteoporosis, pre-existing superior adjacent disc degeneration, prolonged operative time, and increased intraoperative blood loss are independent risk factors for postoperative complications, regardless of the fusion modality employed.

### 4.1. BMI as a Core Risk Factor for Postoperative Complications

The association between elevated BMI and adverse outcomes following lumbar fusion has been consistently reported in the literature [10,23,24]. Finite element modeling studies have confirmed that each unit increase in BMI raises intradiscal pressure by 8–12% and increases facet joint loading by 10–15%, accelerating adjacent segment degeneration and increasing cage migration risk. The increased stress accelerates disc degeneration and compromises the stability of the interbody cage or pedicle screws, thereby increasing the risk of CM and ASD. Moreover, obese patients often exhibit poorer soft tissue quality and impaired wound healing, further contributing to postoperative complications. These findings underscore the importance of preoperative weight management and nutritional optimization for obese patients undergoing lumbar fusion.

### 4.2. Osteoporosis: A Critical Determinant of Fusion Stability

Osteoporosis emerged as a potent risk factor for complications in our study, with osteoporotic patients facing a 5.62–7.62-fold higher risk of CM and ASD. This is attributed to the compromised bone quality in osteoporotic patients, which reduces the pullout strength of pedicle screws and the stability of the interbody cage [25,26]. In PLIF and Hybrid fusion, the interbody cage relies on the endplate bone for initial stability, while PLF depends on the fusion of the posterior elements [5,7]. Osteoporosis weakens the bone–screw and bone–cage interfaces, leading to implant loosening, cage migration, and ultimately, fusion failure. Preoperative osteoporosis screening using DXA and targeted anti-osteoporotic therapy (e.g., bisphosphonates and teriparatide) may improve implant stability and reduce complication rates. Our findings highlight the need for routine osteoporosis assessment and management in patients undergoing lumbar fusion.

### 4.3. Pre-Existing Adjacent Disc Degeneration: A Predictor of ASD

Preoperative superior adjacent disc degeneration was identified as a robust risk factor for postoperative complications, particularly ASD [27,28,29,30]. Patients with advanced Pfirrmann grades (≥IV) had a 2.87–8.04-fold higher risk of complications, consistent with previous studies. The biomechanical changes induced by spinal fusion are the primary driver of ASD: fusion eliminates motion at the treated levels, shifting the mechanical stress to the adjacent segments. In patients with pre-existing disc degeneration, the already compromised discs are unable to withstand the increased stress, leading to accelerated degeneration [30]. Notably, we observed that superior adjacent disc degeneration was a more significant risk factor than inferior degeneration, which may be attributed to the greater mobility of the upper lumbar segments. These findings emphasize the importance of careful radiological evaluation of adjacent segments preoperatively, as patients with advanced degeneration may benefit from more conservative approaches or prophylactic stabilization.

### 4.4. Strengthening Radiological Assessment: Toward Dynamic Evaluation of Spinal Curvature

In addition to static radiographic parameters such as lumbar lordosis angle, recent advances have introduced dynamic, posture-based techniques for quantifying spinal curvature. For instance, a study using 3D posturography demonstrated that preoperative and postoperative changes in lumbosacral spinal curvatures can be reliably measured, providing complementary information to conventional X-ray assessments [31]. Future research integrating 3D posturography or similar technologies could help identify patients at higher risk of postoperative mechanical complications due to suboptimal dynamic spinal alignment.

### 4.5. Surgical Parameters: Operative Time and Intraoperative Blood Loss

Prolonged operative time and increased intraoperative blood loss were identified as independent risk factors for complications [28,30]. Increased intraoperative blood loss contributes to cage migration and adjacent segment degeneration through multiple pathways. First, excessive bleeding indicates extensive soft tissue dissection and endplate injury, which damage the local bone healing microenvironment and reduce the initial stability of bone-cage and bone–screw interfaces. Second, acute blood loss leads to perioperative anemia, impaired tissue oxygenation, and weakened inflammatory regulation, delaying bone fusion and increasing the risk of implant loosening and migration. Third, massive bleeding may require repeated irrigation and suction, disturbing cage position and segmental biomechanical balance, thereby accelerating adjacent segment stress concentration and degeneration. These mechanisms together explain why increased intraoperative blood loss is independently associated with CM and ASD.

### 4.6. Fusion Versus Nonfusion Surgery: Indications and Rationality

The necessity of fusion surgery for LDDs has been widely debated. Nonfusion techniques such as dynamic stabilization preserve segmental motion and may reduce the incidence of ASD, but they lack sufficient stability for patients with severe instability, spondylolisthesis, or multi-segment stenosis. Recent evidence confirms that fusion surgery remains the gold standard for LDDs with obvious instability or deformity [32,33]. For patients with moderate symptoms and good bone quality, nonfusion may be an alternative, but fusion provides more reliable long-term outcomes [34]. Our study focused on fusion modalities because all enrolled patients had indications for fusion, and our results support the importance of risk factor control to improve fusion safety.

### 4.7. Controversial Risk Factors: Age, Gender, and Inflammatory Biomarkers

Contrary to some studies, we found no significant association between age, gender, and postoperative complications [14,35,36,37]. This discrepancy may be attributed to the relatively narrow age range of our study population (predominantly elderly patients) and the homogeneous gender distribution. Regarding inflammatory biomarkers, while the CLR was significantly elevated in the complication group in univariate analysis, it was not identified as an independent risk factor in logistic regression. This suggests that the CLR may be a secondary marker of systemic inflammation rather than a direct cause of CM and ASD. Similarly, PCT and SAA, which are sensitive markers of bacterial infection, showed no association with complications, likely because our study focused on mechanical complications (CM and ASD) rather than infectious complications. These findings highlight the need for modality-specific biomarker research, with a focus on mechanical stress-related markers for CM and ASD.

### 4.8. Clinical Implications and Study Limitations

The present study provides valuable clinical guidance for the prevention and management of postoperative complications following lumbar fusion. Preoperative assessment should include BMI measurement, osteoporosis screening, and detailed radiological evaluation of adjacent segments. For high-risk patients (e.g., obese, osteoporotic, and advanced adjacent disc degeneration), preoperative optimization (e.g., weight loss and anti-osteoporotic therapy) and intraoperative precautions (e.g., minimizing operative time and controlling blood loss) are essential. Additionally, close postoperative follow-up with radiological monitoring is warranted to detect early signs of CM and ASD, allowing for timely intervention.

Despite its strengths, this study has several limitations. This is a single-center retrospective study, which carries inherent selection bias, as patients were treated by a limited group of surgeons with standardized but non-randomized indications. There was no external validation or cohort splitting. Thus, results may not fully generalize to other populations, hospitals, or implant systems. Furthermore, our radiological assessment relied primarily on static X-ray measurements of lumbar lordosis and Pfirrmann grading, without incorporating dynamic, posture-based evaluations such as 3D posturography. Future multi-center prospective studies with internal/external validation and dynamic spinal curvature analysis are needed to confirm our findings and further elucidate the relationship between postoperative sagittal alignment and complications.

## 5. Conclusions

Elevated BMI, osteoporosis, pre-existing superior adjacent disc degeneration, prolonged operative time, and increased intraoperative blood loss are shared independent risk factors for cage migration and adjacent segment degeneration following PLIF, PLF, and Hybrid fusion for lumbar degenerative diseases. Targeted interventions addressing these risk factors may reduce postoperative complication rates and improve patient outcomes. Surgeons should prioritize preoperative optimization and intraoperative precision to mitigate these risks, regardless of the fusion modality employed. Incorporating advanced dynamic assessment tools, such as 3D posturography, into future research protocols may further refine risk stratification and surgical planning.

## Figures and Tables

**Table 1:** Comparison of postoperative complications in the three groups.

Postoperative Complications	PLIF Group	PLF Group	Hybrid Group
Cage Migration	15	NA	6
ASD	65	59	58
Total	80	59	64

**Table 2:** Comparison of intraoperative indexes between normal group and complication group among the three surgical methods.

Indicators	PLIF Postoperative Normal Group, *N* = 80	PLIF Postoperative Complication Group, *N* = 80	Test Statistic	*p*-Value	PLF Postoperative Normal Group, *N* = 93	PLF Postoperative Complication Group, *N* = 59	Test Statistic	*p*-Value	Hybrid Postoperative Normal Group, *N* = 80	Hybrid Postoperative Complication Group, *N* = 64	Test Statistic	*p*-Value
Operation Time (min)	219.0 ± 49.4	230.0 ± 45.2	3.19	<0.01 *	158.1 ± 34.68	176.1 ± 36.47	3.30	<0.01 *	180.3 ± 33.6	205.6 ± 40.5	4.424	<0.01 *
Average Postoperative Drainage Volume (mL)	387.4 ± 71.8	405.4 ± 85.9	0.33	0.73	319.2 ± 90.5	320.7 ± 95.5	0.09	0.92	290.1 ± 86.9	300.8 ± 91.6	1.921	0.057
Intraoperative Blood Loss (mL)	350.17 ± 85.7	399.17 ± 94.7	2.29	0.02	316.8 ± 86.8	351.1 ± 90.5	3.08	<0.01 *	370.75 ± 95.24	408.6 ± 89.2	4.414	<0.01 *

* representing *p* < 0.05, the data results have statistical significance.

**Table 3:** Comparison of biological indexes between the normal group and the complication group after the three types of surgery.

Indicators	PLIF Postoperative Normal Group, *N* = 80	PLIF Postoperative Complication Group, *N* = 80	Test Statistic	*p*-Value	PLF Postoperative Normal Group, *N* = 93	PLF Postoperative Complication Group, *N* = 59	Test Statistic	*p*-Value	Hybrid Postoperative Normal Group, *N* = 80	Hybrid Postoperative Complication Group, *N* = 64	Test Statistic	*p*-Value
CLR	1.81 ± 0.63	2.15 ± 0.71	7.24	<0.01 *	1.68 ± 0.78	2.09 ± 0.67	3.06	<0.01 *	1.77 ± 0.32	2.13 ± 0.38	6.362	<0.01 *
PCT (ug/L)	1.07 ± 0.23	1.17 ± 0.85	0.90	0.37	1.27 ± 0.63	1.36 ± 0.91	1.18	0.24	1.24 ± 0.53	1.16 ± 0.83	1.816	0.071
SAA (mg/L)	8.76 ± 2.23	9.15 ± 3.83	1.23	0.10	8.43 ± 2.63	9.34 ± 3.59	1.56	0.09	7.13 ± 3.54	8.56 ± 3.79	1.793	0.075

* representing *p* < 0.05, the data results have statistical significance.

**Table 4:** Comparison of clinical efficacy between normal group and complication group among the three surgical methods.

Indicators	PLIF Postoperative Normal Group, *N* = 80	PLIF Postoperative Complication Group, *N* = 80	Test Statistic	*p*-Value	PLF Postoperative Normal Group, *N* = 93	PLF Postoperative Complication Group, *N* = 59	Test Statistic	*p*-Value	Hybrid Postoperative Normal Group, *N* = 80	Hybrid Postoperative Complication Group, *N* = 64	Test Statistic	*p*-Value
ODI	65.39 ± 6.56	72.34 ± 4.64	7.68	<0.01 *	64.29 ± 4.56	71.34 ± 5.54	8.71	<0.01 *	62.29 ± 6.16	70.74 ± 4.84	10.864	<0.05
JOA Score	13.42 ± 2.71	10.92 ± 2.45	5.98	<0.01 *	12.22 ± 2.71	9.32 ± 3.01	6.83	<0.01 *	13.29 ± 2.51	10.82 ± 2.55	4.792	<0.05
VAS Score	4.72 ± 2.89	6.59 ± 2.37	6.21	<0.01 *	5.05 ± 2.79	6.92 ± 2.36	4.65	<0.01 *	4.87 ± 2.56	6.78 ± 2.47	6.342	<0.05
SF-36 Score	23.03 ± 5.53	21.04 ± 5.17	4.85	<0.01 *	21.53 ± 4.53	22.04 ± 4.21	0.815	0.42	23.13 ± 5.43	21.54 ± 4.57	1.924	0.059

* representing *p* < 0.05, the data results have statistical significance.

## Data Availability

All the data used in the article can be obtained from the medical record information system of the sixth medical center of PLA general hospital. Any questions or enquiries regarding the present study can be directed to Dike Ruan (ruandikengh@163.com).

## Supplementary Materials

The following supporting information can be downloaded at: https://www.mdpi.com/article/10.3390/jcm15114195/s1, Table S1. Related factors and quantitative assignments; Table S2. Comparison of basic data between normal and complication groups after three surgeries; Table S3. Comparison of clinical indicators between the normal group and the complication group after the three types of surgery; Table S4. Analysis of risk factors for complications after PLIF; Table S5. Analysis of risk factors for complications after PLF; Table S6. Analysis of risk factors for complications after hybrid.
