# Supplementary material for: Risk Factors for Postoperative Complications in Different Fusion Surgical Approaches for Lumbar Degenerative Diseases
Source: J Clin Med. 2026 May 29;15(11):4195. doi: 10.3390/jcm15114195 (PMC13257703; doi:10.3390/jcm15114195)
Supplement: Supplementary file 1 [file jcm-15-04195-s001.zip › jcm-4298830-supplementary.pdf]

Supplemental table S1. Related factors and quantitative assignments

Variable		Assignment	
Y	postoperative complications	none=0	yes=1
X1	gender	female=0	male=1
X2	hypertension	none=0	yes =1
X3	diabetes	none =0	yes =1
X5	hypoproteinemia	none =0	yes =1
X6	insobriety	none =0	yes =1
X7	smoking	none =0	yes =1
X8	overweight	none =0	yes =1
X9	hyperuricemia	none =0	yes =1

Supplemental table S2. Comparison of basic data between normal and complication groups after three surgeries

Indicators	PLIF Postoperative Normal Group N=80	PLIF Postoperative Complication Group N=80	Test Statistic	P Value	PLF Postoperative Normal Group N=93	PLF Postoperative Complication Group N=59	Test Statistic	P Value	Hybrid Postoperative Normal Group N=80	Hybrid Postoperative Complication Group N=64	Test Statistic	P Value
Age (years)	61.59±7.35	63.28±9.23	0.330	0.74	62.29±5.35	63.31±8.32	0.096	0.92	62.49±6.35	64.28±7.12	0.498	0.496
Gender			0.102	0.75			0.563	0.45			0.140	0.708
Male	36(45%)	34(42.5%)			42(54.2%)	23(39.0%)			35(43.7%)	30(37.5%)		
Female	44(55%)	46(47.5%)			51(54.8%)	36(61.0%)			45(56.3%)	34(62.5%)		
BMI (kg/m^2)	22.35±2.89	24.97±3.12	4.38	<0.01*	22.23±3.19	24.89±3.45	5.530	<0.01*	21.95±3.01	24.88±2.49	5.637	<0.01*
BMD	-0.56±0.73	-2.34±0.93	15.3	<0.01*	-0.66±0.88	-2.32±0.65	12.88	<0.01*	-0.77±0.38	-2.30±0.86	15.700	<0.01*
Diabetes			0.250	0.62			0.763	0.15			0.086	0.769
None	72(10.00%)	70(12.5%)			85(91.4%)	53(23.03%)			74(92.5%)	60(93.8%)		
Yes	8(45.00%)	10(87.5%)			8(8.6%)	6(40.13%)			6(7.5%)	4(6.2%)		
Smoking			0.502	0.48			0.161	0.68			0.029	0.865
None	56(70.0%)	60(75.0%)			72(77.4%)	44(74.5%)			59(73.8%)	48(75.0%)		
Yes	24(30.0%)	20(25.0%)			21(22.6%)	15(25.5%)			21(26.2%)	16(25.0%)		
Hypertension			0.625	0.43			3.495	0.06			0.033	0.855
None	62(77.5%)	66(82.5%)			78(83.9%)	42(71.2%)			72(90.0%)	57(89.1%)		
Yes	18(22.5%)	14(17.5%)			15(16.1%)	17(28.8%)			8(10.0%)	7(10.9%)		
Hyperuricemia			5.980	0.01			5.78	0.04*			2.990	0.084
None	64(80.0%)	50(62.5%)			82(88.2%)	43(72.8%)			66(82.5%)	45(70.3%)		
Yes	16(20.0%)	30(37.5%)			11(11.8%)	16(27.2%)			14(17.5%)	19(29.7%)		
insobriety			0.105	0.75			0.002	0.96			0.193	0.661
None	50(62.3%)	48(60.0%)			77(82.8%)	49(83.1%)			60(75.0%)	50(78.1%)		
Yes	30(37.7%)	32(40.0%)			16(17.2%)	10(16.9%)			20(25.0%)	14(21.9%)		
Osteoporosis			10.0	<0.01*			6.930	<0.01			8.413	<0.01*
None	72(90.0%)	56(70.0%)			82(88.2%)	42(71.2%)			74(92.5%)	48(75.0%)		
Yes	8(10.0%)	24(30.0%)			11(11.8%)	17(28.0%)			6(7.5%)	16(25.0%)		
Hypoproteinemia			6.23	0.02*			0.416	0.52			0.474	0.491
None	72(90.0%)	65(18.8%)			75(80.6%)	45(76.3%)			65(81.3%)	49(76.6%)		

Yes	8(10.0%)	15(81.2%)			18(19.4%)	13(23.7%)			15(18.7%)	15(23.4%)		

*representing p<0.05, the data results have statistical significance

Supplemental table S3. Comparison of clinical indicators between the normal group and the complication group after the three types of surgery

Indicators	PLIF Postoperative Normal Group N=80	PLIF Postoperative Complication Group N=80	Test Statistic	P Value	PLF Postoperative Normal Group N=93	PLF Postoperative Complication Group N=59	Test Statistic	P Value	Hybrid Postoperative Normal Group N=80	Hybrid Postoperative Complication Group N=64	Test Statistic	P Value
Type of Degenerative Lumbar Disease			1.22	0.54			1.078	0.58			3.394	0.183
Degenerative Lumbar Spondylolisthesis	24(30.0%)	30(37.5%)			38(40.9%)	20(33.9%)			26(32.5%)	30(46.9%)		
Lumbar Spinal Stenosis	30(37.5%)	30(36.3%)			37(39.8%)	24(40.7%)			34(43.8%)	24(37.5%)		
Lumbar Disc Herniation	26(32.5%)	20(26.3%)			18(19.4%)	15(25.4%)			20(23.8%)	10(15.6%)		
Pfirrmann Grading of Upper Adjacent Disc Degeneration			Fisher	<0.05			4.92	0.08			2.361	0.307
I-II	57(72.5%)	43(52.5%)			70(75.3%)	35(59.3%)			45(52.5%)	29(42.8%)		
III-IV	19(23.8%)	24(28.7%)			17(16.1%)	12(22.0%)			27(40.0%)	24(38.5%)		
V	4（3.8%）	13(18.8%)			8（8.6%）	12(18.6%)			8(7.5%)	13(18.7%)		
Pfirrmann Grading of Lower Adjacent Disc Degeneration			7.88	0.02*			6.12	0.04*			8.12	0.02*
I- II	40(50.0%)	24(30.0%)			69(73.2%)	31(52.5%)			32(40.0%)	32(50.0%)		
III- IV	30(37.5%)	36(45.0%)			17(18.7%)	17(28.8%)			42(52.5%)	20 (31.2%)		
V	10(12.5%)	20(25.0%)			9(8.2%)	11(18.6%)			6（7.5%）	12(18.8%)		
Number of Fused Segments			0.235	0.37			5.77	0.56		2.031		0.362
2	38(47.5%)	30(25.0%)			66(71.0%)	31(52.5%)			45(56.3%)	29(45.3%)		
3	28(35.0%)	40(50.0%)			17(18.3%)	20(33.9%)			26(32.5%)	28(43.8%)		
4	14(17.5%)	10(25.0%)			10(10.8%)	8(13.6%)			9(11.3%)	7(10.9%)		
Lumbar Lordosis Angle (degrees)	44.5±9.2	39.6±10.9	5.78	<0.01*	42.6±8.9	37.6±6.7	3.43	0.01*	47.4±8.9	39.5±7.9	5.707	<0.01*

*representing p<0.05, the data results have statistical significance

Supplemental table S4. Analysis of risk factors for complications after PLIF

Risk Factors	B	SE	Wals	P Value	OR	95%CI Lower Bound	95%CI Upper Bound
BMI	1.09	0.52	4.32	0.03*	1.18	1.05	4.38
BMD	1.08	0.53	4.15	0.041*	.053	0.01	0.18
Hyperuricemia	1.53	7.24	0.04	0.83	4.64	0.28	1.25
Pfirrmann Grading of Upper Adjacent Disc Degeneration	2.08	1.95	1.13	0.01*	8.04	0.17	374.11
Osteoporosis	2.38	1.14	4.37	0.03*	6.86	1.16	81.57
Lumbar Lordosis Angle (degrees)	-0.69	0.27	6.36	0.01*	0.05	1.16	3.42
CLR	1.17	1.56	0.51	0.47	3.08	0.14	66.43
Operation Time (min)	0.005	0.008	0.40	0.01*	1.005	0.98	1.02
Intraoperative Blood Loss (ml)	.019	.025	.577	0.01*	1.019	0.97	1.07
ODI	0.25	0.15	2.41	0.12	0.79	0.58	1.06
JOA Score	-0.08	0.04	3.86	0.05	0.91	0.84	1.00
VAS Score	1.42	3.39	0.17	0.67	4.17	0.05	336.98
SF-36 Score	0.62	3.61	0.03	0.86	1.87	0.04	222.51

*representing p<0.05, the data results have statistical significance

Supplemental table S5. Analysis of risk factors for complications after PLF

Risk Factors	B	SE	Wals	P Value	OR	95%CI Lower Bound	95%CI Upper Bound
BMI	0.25	0.269	0.89	0.04*	1.190	0.76	2.18
BMD	-5.79	2.010	8.31	<0.01*	0.003	0.00	0.15
Hyperuricemia	-0.40	1.419	0.08	0.776	0.668	0.04	10.79
Pfirrmann Grading of Upper Adjacent Disc Degeneration	2.03	1.096	3.43	0.01*	7.627	0.89	65.34
Osteoporosis	1.50	0.649	5.36	0.02*	4.494	1.25	16.03
Lumbar Lordosis Angle (degrees)	-0.65	0.276	5.54	0.07	0.020	1.11	1.39
CLR	2.03	1.096	3.43	0.064	7.627	0.89	65.34
Operation Time (min)	0.01	0.018	0.84	0.01*	1.017	0.98	1.05
Intraoperative Blood Loss (ml)	0.004	0.007	0.37	0.01*	1.004	0.99	1.01
ODI	0.95	0.376	5.58	0.06	1.718	1.01	3.17
JOA Score	-0.66	0.392	2.84	0.092	0.516	0.23	1.11
VAS Score	0.25	0.282	0.81	0.366	1.290	0.74	2.24

*representing p<0.05, the data results have statistical significance

Supplemental table S6. Analysis of risk factors for complications after hybrid

Risk Factors	B	SE	Wals	P Value	OR	95%CI Lower Bound	95%CI Upper Bound
BMI	0.50	0.22	5.16	0.02*	1.142	1.071	2.54
BMD	-4.71	1.26	13.90	<0.01*	1.009	0.001	0.10
Hyperuricemia	3.72	2.06	3.23	0.01*	5.627	1.07	60.34
Pfirrmann Grading of Upper Adjacent Disc Degeneration	1.05	1.54	0.46	0.03*	2.875	0.13	59.80
Osteoporosis	-0.12	0.05	4.68	0.02*	0.03	0.88	0.78
Lumbar Lordosis Angle (degrees)	1.20	1.650	0.53	0.46	3.330	0.13	84.52
CLR	0.02	.017	2.31	0.03*	1.027	0.99	1.06
Operation Time (min)	0.005	.005	0.90	0.02*	1.005	0.99	1.01
Intraoperative Blood Loss (ml)	0.37	.074	25.27	0.07	1.449	1.25	1.67
ODI	-0.42	.136	9.84	0.06	0.652	0.50	0.85
JOA Score	0.46	.131	12.39	0.07	1.584	1.22	2.04

*representing p<0.05, the data results have statistical significance
